# Adjusting MtDNA Quantification in Whole Blood for Peripheral Blood Platelet and Leukocyte Counts

**DOI:** 10.1371/journal.pone.0163770

**Published:** 2016-10-13

**Authors:** Yamilee Hurtado-Roca, Marta Ledesma, Monica Gonzalez-Lazaro, Raquel Moreno-Loshuertos, Patricio Fernandez-Silva, Jose Antonio Enriquez, Martin Laclaustra

**Affiliations:** 1 CIBERESP, Department of Preventive Medicine and Public Health, School of Medicine, Universidad Autónoma de Madrid, Madrid, Spain; 2 Centro Nacional de Investigaciones Cardiovasculares Carlos III, Madrid, Spain; 3 Boca Raton Clinical Research Global Peru, Lima, Peru; 4 Instituto Aragonés de Ciencias de la Salud, Zaragoza, Spain; 5 Dpto. de Bioquímica y Biología Molecular y Celular, Universidad de Zaragoza, Zaragoza, Spain; 6 Department of Epidemiology, St. Louis University, St Louis, Missouri, United States of America; Vanderbilt University Medical Center, UNITED STATES

## Abstract

Alterations of mitochondrial DNA copy number (mtDNAcn) in the blood (mitochondrial to nuclear DNA ratio) appear associated with several systemic diseases, including primary mitochondrial disorders, carcinogenesis, and hematologic diseases. Measuring mtDNAcn in DNA extracted from whole blood (WB) instead of from peripheral blood mononuclear cells or buffy coat may yield different results due to mitochondrial DNA present in platelets. The aim of this work is to quantify the contribution of platelets to mtDNAcn in whole blood [mtDNAcn(WB)] and to propose a correction formula to estimate leukocytes' mtDNAcn [mtDNAcn(L)] from mtDNAcn(WB). Blood samples from 10 healthy adults were combined with platelet-enriched plasma and saline solution to produce artificial blood preparations. Aliquots of each sample were combined with five different platelet concentrations. In 46 of these blood preparations, mtDNAcn was measured by qPCR. MtDNAcn(WB) increased 1.07 (95%CI 0.86, 1.29; p<0.001) per 1000 platelets present in the preparation. We proved that leukocyte count should also be taken into account as mtDNAcn(WB) was inversely associated with leukocyte count; it increased 1.10 (95%CI 0.95, 1.25, p<0.001) per unit increase of the ratio between platelet and leukocyte counts. If hematological measurements are available, subtracting 1.10 the platelets/leukocyte ratio from mtDNAcn(WB) may serve as an estimation for mtDNAcn(L). Both platelet and leukocyte counts in the sample are important sources of variation if comparing mtDNAcn among groups of patients when mtDNAcn is measured in DNA extracted from whole blood. Not taking the platelet/leukocyte ratio into account in whole blood measurements, may lead to overestimation and misclassification if interpreted as leukocytes' mtDNAcn.

## Introduction

Mitochondria are intracellular organelles involved in energy production through the process of oxidative phosphorylation (OXPHOS) that have their own genome (mtDNA), distinct from that in the cell nucleus (nDNA). Although the mitochondrial mass per cell varies with cell type and metabolic state, each cell type typically contains a fairly constant amount of mitochondria and accordingly, the number of copies of the mitochondrial genome is also constant, as it is linked to mitochondrial mass[1]. Each mitochondrion contains between 2 and 10 copies of its genome[2]. High-energy requiring cells, such as muscle and neurons, contain a large number of mtDNA copies, while low-energy requiring cells, such as spleen and endothelial cells, contain fewer copies[3–5]. The amount of mtDNA per cell, or mtDNA copy number (mtDNAcn), can be expressed as a ratio of mtDNA to nDNA copies, i.e. using nDNA as reference, assuming that all quantified cells are nucleated and diploid[6]. Nowadays, quantitative real-time PCR (qPCR) is a suitable method to quantify mtDNAcn[7–9].

Either reduction or increase in the biogenesis or availability of mitochondria in the cells may be markers of primary mitochondrial pathology or of systemic pathology that affects mitochondrial biology. Although mtDNAcn can be studied in any tissue, blood is one of the most commonly used, as samples can be easily obtained. Alterations of mtDNAcn in the blood may be associated to primary mitochondrial disorders[10,11], which are sometimes associated to primary genetic mutations[12,13], but have also been linked to cardiac dysfunction[14], carcinogenesis and cancer progression[15,16], HIV infection[17,18], diabetes[19–21], and microalbuminuria[22].

For the study of mtDNAcn, DNA is usually obtained from peripheral blood mononuclear cells (PBMC). In such samples, platelet contamination may lead to overestimation of mtDNAcn measurements[23] since one platelet contains around 1.6 molecules of mtDNA on average but no nuclear DNA[24]. Timmermans et al.[18] reported that contaminating platelets did not influence the results if their total number is below 5 times the number of PBMC. Most large-scale epidemiological studies have DNA samples available, but in many cases they have been extracted from whole blood and not from PBMC[25–27]. Healthy subjects usually have 14–90 times more platelets than leukocytes in peripheral blood[28]. Therefore, a pool of PBMCs’, granulocytes’, and platelets’ mtDNA is present in those samples, which could affect mtDNAcn quantification. In fact, many studies have been performed using mtDNAcn data measured in whole blood, and although they were able to establish associations with morbid processes such as cancer[15,16], non-Hodgkin lymphoma[29,30], Huntington's disease[31], diabetes[19–21], and microalbuminuria[22], considering the effect of platelets could improve the discovery process.

The aims of this work are to describe the effect of platelet count in mtDNAcn in whole blood, to evaluate the extent of mtDNAcn overestimation and misclassification on real epidemiological data, and to propose a correction formula to estimate leukocytes’ mtDNAcn [mtDNAcn(L)] from whole blood mtDNAcn [mtDNAcn(WB)], if hematological measurements of the original sample are available.

## Materials and Methods

### Design

MtDNAcn was measured in several artificial blood preparations that were experimental combinations of blood samples with platelet-enriched plasma and saline solution so that each sample was combined with five different platelet concentrations. The coefficients that relate hematological parameters with mtDNAcn, according to a theoretical formulation framework, were estimated statistically. Afterwards, mtDNAcn data from an observational study was used to describe the differences in value and subject classification between raw mtDNAcn measurements in whole blood and values corrected to approximate mtDNAcn(L) using the previously estimated coefficients.

### Samples

Fasting whole blood samples from 10 randomly selected (8 men and 2 women) participants of the Aragon Workers’ Health Study[32] whose complete blood count was inside healthy ranges were used to produce the artificial blood preparations for the experiments. The samples were collected in 6 ml EDTA-K2 tubes (BD Vacutainer). The Aragon Workers’ Health Study, AWHS, is a longitudinal study of healthy middle-aged workers in a large car factory in Spain, and data from the whole cohort with available mtDNAcn (N = 3389) was used to compare mtDNAcn(WB) and calculated mtDNAcn(L). All participants were informed about the AWHS and the potential research uses of their samples and they signed a written informed consent approved by the Clinical Research Ethics Committee of Aragon (CEICA).

### Experimental protocol

After measuring blood cells count, each participant’s sample was processed to obtain 5 derived artificial blood preparations, so that each one contained a different proportion of leukocytes and platelets. DNA was subsequently extracted from the original samples and the derived preparations, and mtDNAcn was measured in the extracted DNA.

### Blood preparations

For each participant, six 50 μl aliquots of whole blood were created and reserved. From the rest of the sampled blood, platelets were isolated to obtain platelet-enriched plasma. In brief, blood was centrifuged at 100 x g for 15 min at room temperature. The upper layer was transferred into a new tube and was centrifuged once more at 200 x g for 10 min at room temperature. This supernatant, containing the platelets (platelet enriched plasma, PEP), was saved to be combined in different proportions with saline solution (Table 1) and it was added to five of the whole blood reserved aliquots. Blood counts were obtained from the preparations. Samples and preparations were kept at -80°C until DNA extraction.

**Table 1:** Proportions for the blood preparations.

	Preparation number				
	P1	P2	P3	P4	P5
Blood (μl)	50	50	50	50	50
Saline solution (μl)	400	387.5	350	200	0
Platelet-enriched plasma (PEP) (μl)	0	12.5	50	200	400
Ratio of PEP volume with respect to P2	-	1x	4x	16x	32x

### Blood count

Blood samples and preparations were analyzed in a COULTER ACT 5diff analyzer (Beckman Coulter) to determine platelet and leukocyte counts. Internal quality controls of three levels were measured before the daily work. An external quality control from the Spanish Association of Pharmaceutical Analysts (AEFA) was processed every 4-months.

### DNA extraction and Quantitative Polymerase Chain Reaction assays for mtDNA

For the experimental part, DNA was extracted manually from the blood samples and blood preparations with phenol-chloroform, according to the protocol described by Marcuello et al.[33]. Concentration and purity of DNA were measured with a Nanovue spectrophotometer (Thermo Fisher Scientific, USA) and DNA dilutions at 2 ng/μl were prepared for the analyses. For the AWHS cohort samples, DNA was extracted with the AutoGenFlex 3000 extractor, using the FlexiGene DNA kit (Qiagen), which is based on alcoholic precipitation. Similarly, DNA dilutions at 2 ng/μl were prepared for the subsequent mtDNAcn measurement.

Quantification of mtDNAcn relied on quantitative real-time polymerase chain reactions (qPCR) based on SYBR Green assays and performed in an ABI PRISM®7900 Real-Time PCR System (Applied BioSystems). In a single 384-well reaction plate, two sets of 10 μl reactions were performed in separate reaction wells, one for the mitochondrial cytochrome oxidase II gene (*MT-CO2*), and one for the nuclear gene for the A subunit of succinate dehydrogenase (*SDHA*), producing amplicons of 69 and 72 bp respectively. Each reaction contained 5 ng of DNA and 400 nM of each primer. Primer sequences and positions are indicated in Table A in S1 File. The samples as well as a negative control and a 7-point 1:10 dilution curve of a standard (described below) were analyzed in triplicate. Conditions used for amplification were as follows: one cycle of 50°C for 2 min, one cycle of 95°C for 10 min, 40 cycles of 95°C for 1 sec. and 60°C for 20 sec., one cycle of 60°C for 15 sec and 1 cycle of 95°C for 15 sec. To accommodate all the measurements in a single reaction plate we measured only 46 out of the 50 preparations (we left out four P3 preparations).

The standard curve, which contained an equal number of copies for both amplicons (see below), was used as a reference for absolute quantification of each gene using the threshold cycle number (Cq) method. The thresholds were obtained by the default second derivate method on ABI PRISM (Sequence Detection System, SDS 2.4 software). The ratio of the absolute quantity of *MT-CO2* to that of *SDHA* (mtDNA copy number—mtDNAcn) was used as a measurement of half of the number of copies of mtDNA in leukocytes. We use the term mtDNAcn(WB) to refer to the direct (uncorrected) measurements performed in whole blood samples or blood preparations in contrast to mtDNAcn(L) which would be obtained from measuring in leukocytes or derived with our correction formula (see below).

### Standard

A PCR standard was created by ligating one copy of the amplicon for *MT-CO2* and one copy of the amplicon for *SDHA*[34]. A longer amplicon (224 bp) for the *MT-CO2* gene (Table A in S1 File), which included the region used for qPCR determination, was used to allow an adequate discrimination of the correct ligation products after PCR amplification. The sequence of the PCR standard was verified by Sanger sequencing. The standard was prepared by performing six 10-fold dilutions from an initial concentration of 0.1ng/μl.

### Formulation framework

In whole blood, the number of copies of mtDNA (*n*_*mtDNA*_) is the sum of the number of copies of mtDNA per leukocyte (*mtDNA*_*L*_) multiplied by the count of leukocytes (*n*_*L*_) plus the number of copies of mtDNA per platelet (*mtDNA*_*P*_) multiplied by the count of platelets (*n*_*P*_).

$$n_{mtDNA}={mtDNA}_{L}\cdot n_{L}+{mtDNA}_{P}\cdot n_{P}$$

Similarly, the number of copies of nDNA (*n*_*nDNA*_) is the number of copies of nDNA per leukocyte (*nDNA*_*L*_), assuming 2 in diploid cells (*nDNA*_*L*_ = 2), multiplied by the count of leukocytes (*n*_*L*_).

$$n_{nDNA}={nDNA}_{L}\cdot n_{L}$$

The ratio of mtDNA (*mtDNAcn*) is calculated by dividing the number of copies of mtDNA (*n*_*mtDNA*_) by the number of copies of nDNA (*n*_*nDNA*_). Given that,

$$mtDNAcn=\frac{n_{mtDNA}}{n_{nDNA}}\;and\;{nDNA}_{L}=2,$$

in whole blood,

$$mtDNAcn(WB)=\frac{{mtDNA}_{L}\cdot n_{L}}{2\cdot n_{L}}+\frac{{mtDNA}_{P}\cdot n_{P}}{2\cdot n_{L}},$$

while in leukocytes,

$$mtDNAcn(L)=\frac{{mtDNA}_{L}}{2}.$$

Replacing *mtDNAcn*(*L*) appropriately, we obtain:

$$mtDNAcn(WB)=mtDNAcn(L)+\frac{{mtDNA}_{P}}{2}\cdot \frac{n_{P}}{n_{L}}$$

$$mtDNAcn(WB)=mtDNAcn(L)+K\cdot \frac{n_{P}}{n_{L}}$$

The term $\frac{{mtDNA}_{P}}{2}=K$ represents the factor that multiplies the ratio between platelets and leukocyte counts to calculate the excess value of *mtDNAcn*(*WB*) with respect to *mtDNAcn*(*L*). We estimated the factor (*K*) by means of a statistical model:

$$mtDNAcn(WB)∼\beta_{0}+\beta_{1}\cdot \frac{n_{P}}{n_{L}}$$

in which estimating β_0_ will provide an estimation for *mtDNAcn*(*L*), assumed constant for our samples, and estimating β_1_ will provide an estimation for $K=\frac{{mtDNA}_{P}}{2}$.

Thus, the formula that we propose for correcting *mtDNAcn*(*WB*) is:

$$mtDNAcn(L)=mtDNAcn(WB)-K\cdot \frac{n_{P}}{n_{L}}$$

### Statistical analysis

Data are presented as mean and standard deviation (SD). All statistical analyses were performed with the statistical package R (version 3.0.2)[35]. The coefficients for the association between leukocytes and platelets with mtDNAcn(WB) were determined using linear regression analysis. We built multivariate regression models to determine the effect of platelet and leukocyte counts on mtDNAcn(WB). We first modeled the association between mtDNAcn(WB) and platelets count; then we performed models to determine to what extent the inverse of leukocyte count was a confounding factor or an effect modifier, so as to check whether empirical data matched our formulation framework. Given that our 46 preparations were originated from 10 samples we verified our estimation using multilevel mixed-effects models: a model with common slope and random intercept terms (model 1), one with correlated random intercept and slope (model 2), and one with uncorrelated random intercept and slope (model 3). The statistical significance threshold was set at p<0.05.

We calculated mtDNAcn(L) from mtDNAcn(WB) for the AWHS participants using our correction formula. We described the mean, standard deviation and percentiles, as well as cross-classification of participants in quintiles of both values. We report the percentage of participants that were classified in a different quintile by each value.

## Results

The hematological values of the primary samples used for the experiment were within normal limits (Table 2). In them, the mean (standard deviation) of mtDNAcn(WB) was 97.22 (28.24).

**Table 2:** Hematological results from primary blood samples (n = 10) that were used to derive blood preparations.

	Mean	Standard Deviation(SD)	Minimum value	Maximum value
**White Blood Cells (10**^**3**^**/μL)**	7.56	1.76	5.3	10.8
**Red Blood Cells (10**^**6**^**/μL)**	4.72	0.38	4.02	5.4
**Hemoglobin (g/dL)**	14.82	1.16	13	17
**Hematocrit (%)**	43.98	3.47	38.2	50.3
**Mean Cell Volume (fL)**	93.2	3.58	88	100
**Mean Cell Hemoglobin (pg)**	31.46	1.02	29.7	33.3
**Mean Corpuscular Hemoglobin Concentration (g/dL)**	33.71	0.27	33.2	34
**Red Cell Distribution Width (%)**	12.61	0.58	11.7	13.4
**Platelet count (10**^**3**^**/μL)**	234.1	38.23	190	295
**Mean Platelet Volume (fL)**	9.25	0.37	8.6	9.9
**Plateletcrit (%)**	0.37	0.48	0.17	1.75
**Platelet Distribution Width (fl)**	15.59	1.2	13	16.5
**Neutrophils (%)**	50.22	5.28	42.3	59.3
**Lymphocytes (%)**	39.72	5.08	33.1	48.9
**Monocytes (%)**	6.68	1.19	4.8	8.2
**Eosinophils (%)**	2.49	0.59	1.2	3.2
**Basophils (%)**	0.89	0.32	0.6	1.5
**Abnormal Lymphocytes (%)**	1.09	0.44	0.5	2
**Immature Cells (%)**	0.69	0.18	0.4	1
**mtDNAcn(WB)**	97.22	28.24	54.29	149.6

mtDNAcn(WB): mitochondrial DNA copy number in whole blood

**Fig 1:**
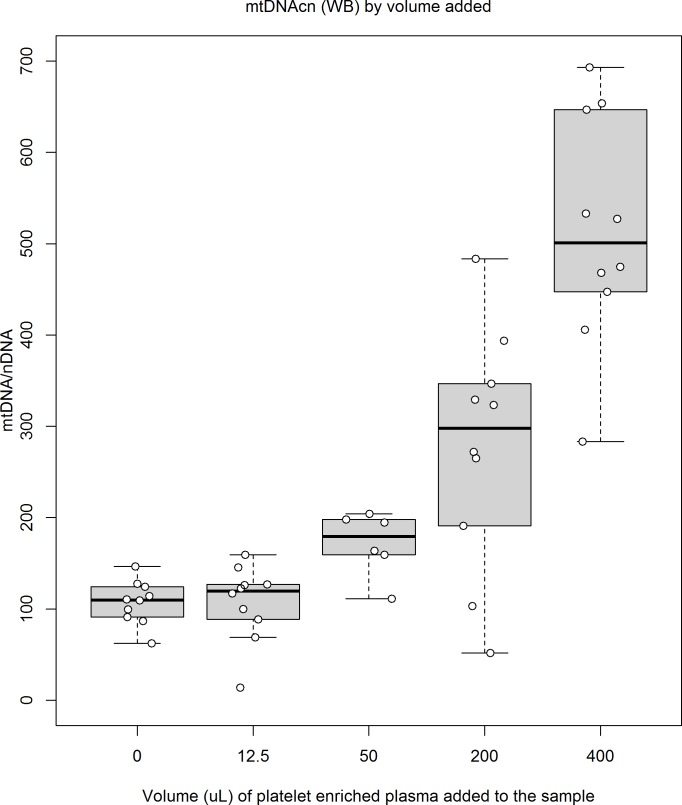
MtDNAcn(WB) of blood preparations supplemented with different amounts of platelet-enriched plasma and saline solution. All preparations combined 50 uL of blood, plus platelet-enriched plasma and additional saline solution to reach a volume of 450 uL. Each box corresponds to a different combination (p1—p5, see Table 1) and summarizes data from 10 preparations (one from each original blood sample) except in p3 (50 uL of platelet-enriched plasma), which only summarizes 6 preparations. Altogether, data from 46 preparations are shown. Individual mtDNAcn(WB) for each preparation are plotted as white circles.

In the blood preparations supplemented with different amounts of platelet-enriched plasma (46 data points), mtDNAcn(WB) increased 1.01 (95%CI 0.84, 1.17; p<0.001) per μl of platelet-enriched plasma added (Fig 1). The mtDNAcn(WB) increased 1.07 (95%CI 0.86, 1.29; p<0.001) per 10^3^ platelets and it also showed an inverse association with leukocyte count (Table 3).

**Table 3:** Coefficients of multivariate regression models for mtDNAcn(WB) measured in blood preparations (n = 46).

	Model 1	Model 2	Model 3	Model 4	Model 5	Model 6
**Platelets (change per 10**^**3**^ **platelets)**	1.07*		1.14*	1.15*	-0.76	
**Leukocytes (change per 10**^**3**^ **leukocytes)**		-132.8	-283.00*			
**1/Leukocytes (change per 1/10**^**3**^ **leukocytes)**				225.08*	21.02	
**Platelets/Leukocytes (change per ratio unit)**					1.79*	1.10*

The table cells show the coefficients of the predictors in the left column.

*p < 0.001, otherwise p > 0.05

Model 1: mtDNAcn(WB) modeled from platelets count

Model 2: mtDNAcn(WB) modeled from leukocytes count

Model 3: mtDNAcn(WB) modeled from platelets and leukocyte counts

Model 4: mtDNAcn(WB) modeled from platelets count and 1/leukocytes

Model 5: mtDNAcn(WB) modeled from platelets count, 1/leukocytes and their interaction (which matches the correction term of the formulation framework)

Model 6: mtDNAcn(WB) modeled from platelets/leukocytes (correction term of the formulation framework)

mtDNAcn: mitochondrial DNA copy number, WB: whole blood

**Fig 2:**
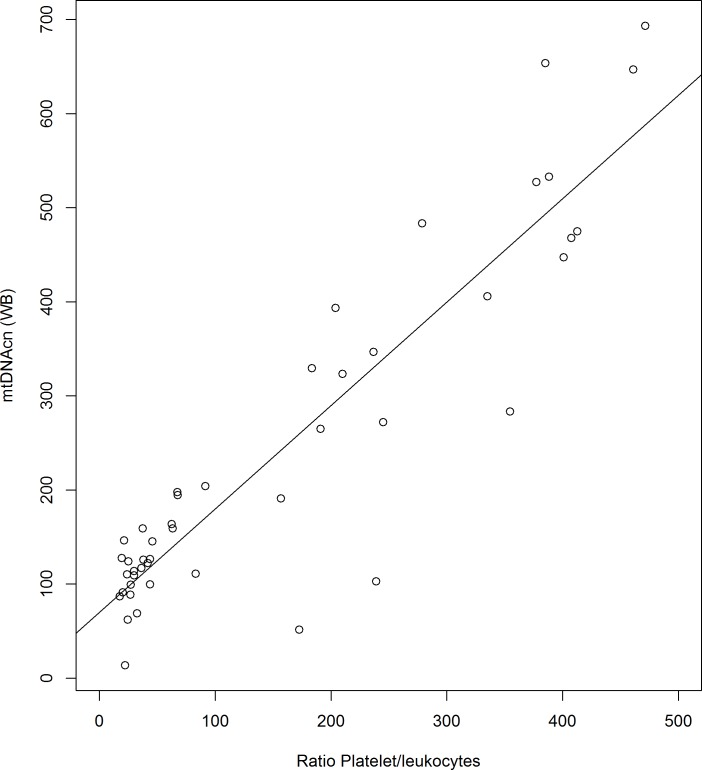
Scatter plot of the change of mtDNAcn(WB) per unit increase of the platelets/leukocyte ratio. This scatter plot shows the change of mtDNAcn(WB) per unit increase of the platelets/leukocyte ratio. Data from 46 preparations are shown. Individual mtDNAcn(WB) for each preparation are plotted as white circles. The line represents the fit of a linear model (see Table 3, Model 6). mtDNAcn(WB): mitochondrial DNA copy number measured in whole blood.

We built multivariate regression models to evaluate the influence of the platelets and leukocytes on mtDNAcn(WB), and we found a significant interaction between platelets and 1/leukocytes that was the only statistically significant term in that model for mtDNAcn(WB)(Table 3), consistent with the correction term that our formulation identified as determinant of the mtDNAcn(WB)(See Formulation framework). According to our formulation framework, with a simple regression model (Table 3 - Model 6) we estimated that mtDNAcn(WB) changed 1.10 (95%CI 0.95, 1.25, p<0.001) per unit increase of the platelets/leukocyte ratio (Fig 2). We also estimated this influence with multilevel models, which took into account the structure of our experiment (preparations derived from 10 different samples), and all models yielded similar coefficients (Table B in S1 File).

In the AWHS study (n = 3389) mtDNAcn(WB) was 68.25 (SD: 15.37) being higher in women than men and the ratio platelets/leukocytes, 33.08 (SD: 9.94) (Table 4). After applying the correction formula with a K factor of 1.1, as calculated from the analysis above, the corrected mtDNAcn(L) was 31.86 (SD: 17.6)(Table 4). In addition, the mtDNAcn quintile of each participant is different depending on whether WB or L value is used (Table C in S1 File). Indeed, 54% of participants are classified in a different quintile by each measurement (although only 15% are classified in quintiles farther away than the neighboring one), suggesting that each parameter conveys different information, and, given the procedure used to calculate mtDNAcn(L), we presume that this measurement will be less dependent on hematological parameters.

**Table 4:** Platelet count, Leukocyte count and mtDNAcn(WB) distributions in all subjects from the AWHS cohort study (n = 3389).

	Overall (n = 3389)					Male (n = 3136)					Female (n = 252)				
	Mean	SD	P5	P50	P95	Mean	SD	P5	P50	P95	Mean	SD	P5	P50	P95
**Platelets count (10**^**3**^**/μL)**	231.16	54.68	152	227	324	229.72	53.9	152.00	224.00	322.00	248.97	61.04	152.40	247.50	354.00
**Leukocytes count (10**^**3**^**/μL)**	7.34	1.96	4.74	7.00	11.00	7.37	1.95	4.80	7.10	11.00	7.05	2.03	4.40	6.80	11.10
**Platelets/Leukocytes**	33.08	9.94	19.20	31.88	51.55	32.74	9.75	19.17	31.62	50.63	37.32	11.21	20.55	36.85	57.24
**mtDNAcn(WB)**	68.25	15.37	46.54	66.43	95.54	67.97	15.35	46.50	66.14	95.16	71.68	15.32	47.46	71.48	99.54
**mtDNAcn(L)**	31.86	17.6	5.37	31.06	60.84	31.96	17.59	5.37	31.06	60.86	30.63	17.75	5.35	30.73	59.64

mtDNAcn: mitochondrial DNA copy number, WB: whole blood, L: leukocytes, SD: standard deviation, P5: percentile 5^th^, P50: percentile 50^th^, P95: percentile 95^th^

## Discussion

In our study, mtDNAcn was measured in whole blood as the ratio between mtDNA and nDNA using quantitative real-time polymerase chain reaction (qPCR) to study the influence of platelet count on mtDNAcn. We compared five sets of artificial blood preparations with different platelet concentrations and observed that the mtDNAcn did not only depend on platelet count but also on the platelets/leukocytes ratio.

We found a significant influence of the number of platelets on mtDNAcn quantification. Similarly, Banas et al.[36] and Andreu et al.[37] found significantly different values when mtDNAcn was measured in whole blood with respect to the measurements performed in PBMCs and buffy coat. Urata et al.[24] showed that platelet contamination, a technical limitation of PBMCs separation, caused large overestimation errors and acted as a source of variation: average±SD mtDNAcn measured in 11 samples of PBMCs decreased from 269±51 to 146±14 after platelet depletion through washing.

The main step forward in our study with respect to previous approaches[23,24,36,37] to the problems derived from the presence of platelets is that we introduce leukocyte count as an essential factor to take into account and contextualize this knowledge for measuring mtDNAcn in whole blood. Dealing with platelets contamination of PBMC samples, Timmermans et al. highlighted that when the number of contaminant platelets surpassed five times the number of cells in the sample, measured mtDNAcn differed from that read on pure PBMC samples[18]. The experiment added platelets to PBMC in fixed ratios and pursued the description of a reasonable threshold for contamination. The approach emphasized that leukocyte count plays a role in the bias introduced by platelets. That safe threshold for ignoring platelets’ influence is not an option for measurements based on whole blood where platelets are usually 30 times the number of leukocytes. Thus, a correction formula is needed for the platelet bias in whole blood mtDNAcn measurements.

Knez et al. measured mtDNAcn in buffy coat in a sample of Flemish population to describe correlations with clinical variables[38]. Consistently with the mathematics of mtDNAcn(WB) which also affects measurements in buffy coat, this study found that after age and sex, the most outstanding clinical variables that correlated with mtDNAcn were platelets count and leukocyte count, the latter with an inverse relationship. The authors concluded that future epidemiological studies should adjust for these variables. Nonetheless, in that study leukocyte count was interpreted as a measurement of systemic inflammation which accordingly opened questions on the association of inflammation with mtDNAcn. This justifies the urgent need to clarify the nature of mtDNAcn(WB) vs. that of mtDNAcn(L) and to provide means to factor out associations arising purely from blood composition, like our proposed formula.

MtDNAcn has been analyzed in whole blood in several clinical situations: chronic diseases such as breast cancer, diabetes mellitus or Huntington´s disease were associated with mtDNA depletion[16,19,30], while others such as non-Hodgkin lymphomas were associated with mtDNA increase[29,30]. It is essential to discriminate whether the clinical associations found in studies performed in whole blood depend on true modifications of the mtDNAcn(L) or on the hematological modifications that may be associated with each disease. Given that in epidemiological studies whole blood is one of the most available samples, we have proposed a correction formula in order to estimate mtDNAcn(L) from mtDNAcn(WB). We applied this correction to AWHS data, showing that mtDNAcn(WB) doubles estimated mtDNAcn(L). This ratio of differences is similar to those reported by Andreu et al.[37] and Urata et al.[24]. The latter also described a decrease in mtDNAcn variability, that we do not observe, but Urata’s study was based on 11 samples while we applied our correction in thousands. In addition, we showed that a non-ignorable amount of the observed mtDNAcn(WB) variation might be due to the differences in hematological values among subjects, as they were classified in different quintiles depending on the measurement used and this might hamper finding the biological and clinical meanings of mtDNAcn if platelets and leukocyte counts are ignored, supporting the use of the correction formula when only whole-blood derived DNA is available. Moreover, several studies reported differences in mtDNA copy number between women and men. Lee et al.[39] showed higher peripheral blood mtDNA copy number in healthy young women compared with men. Recently, Knez et al.[38] reported the same sex difference in general population using buffy coat without platelet depletion. According to our findings, these differences could be partly explained by hematological differences by sex, like the higher number of platelets that women have[40] and further analysis of these sex differences on mtDNAcn taking hematology into account might be clarifying.

In addition to this formula that allows correction for platelet presence in the original sample, there are several aspects that still need to be standardized in the technique of mtDNAcn measurement. Currently, methods for mtDNAcn quantification vary across laboratories and there are several pre-analytical and analytical factors that can affect the final measurement: sample conservation before DNA extraction, time between blood drawing and cell separation, anticoagulant used, DNA extraction method, PCR inhibitors, and DNA quantification method[37]. Until standardization of these variables is achieved, reference values should be established for each measurement protocol and study results should be compared and combined only with extreme caution.

The experimental design allowed comparing several platelet concentrations in preparations derived from blood from the same subject effectively factoring out biological variability to a great extent. Thus, most of the variation observed could be considered technical variation. Therefore, the effective sample size is 46, with data for 5 experimental conditions with 10 replicas (preparations) for almost each condition. These results are a starting point for further expanding this knowledge that will require measuring also natural variation in the platelets and leukocytes counts and mtDNAcn. Those measurements will need the evaluation of a larger sample, as well as double measurements of mtDNAcn in both leukocytes and/or PBMCs and whole blood. This work also has some limitations: Our formula estimates mtDNAcn in blood leukocytes as a whole, which include PBMCs and granulocytes and because of that the estimations cannot be interpreted as a direct replacement of PBMCs’ mtCNDcn. We did not do the parallel extraction of leukocytes and/or PBMCs, which would have provided more details in this regard. Also, DNA extraction was accomplished from the preparations, which were not regular blood, but 1/9 dilutions (Table 1), but this is unlikely to have affected the results as DNA was normalized in concentration after extraction. The range of platelets/leukocytes in the artificial preparations spanned from 20 to 450 while the normal physiological range of this ratio only includes the lower tail of that range. Finally, the AWHS study did not extract leukocytes and/or PBMCs samples so we could only estimate the potential benefit in bias correction using our proposed formula, but not verify the formula itself.

## Conclusions

In conclusion, both platelet and leukocyte counts from the sample are important for comparing the mtDNA content between different patients when mtDNA copy number is estimated from whole blood. Not taking the platelet/leukocyte ratio into account may lead to overestimation of leukocyte mtDNA content and misclassification, which may obscure the meaning of its variation as the non-corrected value depends on hematological differences to a non-ignorable extent. We propose a correction formula that may be used to mitigate this problem. This formula's coefficients are not final and future research will help in refining and validating them.

## Supporting Information

S1 File Supplemental Data. **Table A,** qPCR primers sequences. **Table B,** Multilevel mixed-effect models for mtDNAcn(WB) measured in blood preparations (n = 46). MODEL 1: Common slope and random intercept (per sample) MODEL 2: Correlated random intercept and slope (per sample) MODEL 3: Uncorrelated random intercept and slope (per sample) mtDNAcn: mitochondrial DNA copy number, WB: whole blood. **Table C,** Reclassification of AWHS cohort study subjects (n = 3389) between mtDNAcn(WB) quintiles and mtDNAcn(L) quintiles.
(PDF)
