# Supplementary material for: Adjusting MtDNA Quantification in Whole Blood for Peripheral Blood Platelet and Leukocyte Counts
Source: PLoS One. 2016 Oct 13;11(10):e0163770. doi: 10.1371/journal.pone.0163770 (PMC5063275; doi:10.1371/journal.pone.0163770)
Supplement: S1 File — Table A, qPCR primers sequences. Table B, Multilevel mixed-effect models for mtDNAcn(WB) measured in blood preparations (n = 46). MODEL 1: Common slope and random intercept (per sample) MODEL 2: Correlated random intercept and slope (per sample) MODEL 3: Uncorrelated random intercept and slope (per sample) mtDNAcn: mitochondrial DNA copy number, WB: whole blood. Table C, Reclassification of AWHS cohort study subjects (n = 3389) between mtDNAcn(WB) quintiles and mtDNAcn(L) quintiles. (PDF) [file pone.0163770.s001.pdf]

SUPPLEMENTAL DATA (S1 File)

Table A: qPCR primers sequences

Gene	Strand	Position	Length (bp)	Sequence	Amplicon length (bp)
SDHA	Plus/Plus	223475-223495	21	5'-TCTCCAGTGGCCAACAGTGTT-3'	
(NC_000005.10)	Plus/Minus	223546-223527	20	5'-GCCCTCTTGTTCCCATCAAC-3'	72
MT-CO2	Plus/Plus	7858-7877	20	5'-CGATCCCTCCCTTACCATCA-3'	
(NC_012920.1)	Plus/Minus	7926-7904	23	5'-CCGTAGTCGGTGTACTCGTAGGT-3'	69
	Plus/Minus	8081-8062	20	5'-GGACAGCTCATGAGTGCAAG-3'	224

Y. Hurtado-Roca, M. Ledesma, M. González-Lázaro, R. Moreno-Loshuertos, P. Fernández-Silva, J.A. Enriquez, M. Laclaustra. Adjusting mtDNA quantification in whole blood for peripheral blood platelet and leukocyte counts. PLOS ONE. 2016

Table B: Multilevel mixed-effect models for mtDNAcn(WB) measured in blood preparations (n=46)

	MODEL 1	MODEL 2	MODEL 3
**Generalized Akaike information criterion (AIC)**	528.10	523.32	522.49
**Change in mtDNAcn(WB) per unit change in Platelet/Leukocytes ratio**	1.10 (0.97, 1.22)	1.07 (0.91, 1.23)	1.07 (0.89, 1.25)

MODEL 1: Common slope and random intercept (per sample)

MODEL 2: Correlated random intercept and slope (per sample)

MODEL 3: Uncorrelated random intercept and slope (per sample)

mtDNAcn: mitochondrial DNA copy number, WB: whole blood

Table C: Reclassification of AWHS cohort study subjects (n=3389) between mtDNAcn(WB) quintiles and mtDNAcn(L) quintiles

Quintiles mtDNAcn(WB)	Quintiles mtDNAcn(L)				
	[lowest,17.6] n=678	(17.6,27.2] n=678	(27.2,35.2] n=677	(35.2,45.4] n=678	(45.4, highest] n=678
[lowest,55.6] n=678	375	221	72	10	0
(55.6,62.9] n=678	175	220	189	93	1
(62.9,70.3] n=677	89	130	230	188	40
(70.3,79.7] n=678	31	80	140	258	169
(79.7, highest] n=678	8	27	46	129	468
